# Postpartum breast involution reveals regression of secretory lobules mediated by tissue-remodeling

**DOI:** 10.1186/bcr3633

**Published:** 2014-03-28

**Authors:** Sonali Jindal, Dexiang Gao, Pat Bell, Grethe Albrektsen, Susan M Edgerton, Christine B Ambrosone, Ann D Thor, Virginia F Borges, Pepper Schedin

**Affiliations:** 1 Department of Cell & Developmental Biology, Oregon Health & Science University, 3181 SW Sam Jackson Park Road, Portland, OR 97239, USA; 2 Division of Medical Oncology, School of Medicine, University of Colorado Anschutz Medical Campus, 12801 E 17th Ave, Aurora, CO 80045, USA; 3 Department of Pediatrics, School of Medicine, University of Colorado Anschutz Medical Campus, 12801 E 17th Ave, Aurora, CO 80045, USA; 4 Unit for Applied Clinical Research, Department of Cancer Research and Molecular Medicine, Faculty of Medicine, Norwegian University of Science and Technology, P.B. 8905, 7491 Trondheim, Norway; 5 Department of Pathology, School of Medicine, University of Colorado Anschutz Medical Campus, 12801 E 17th Ave, Aurora, CO 80045, USA; 6 Department of Cancer Prevention and Control, Roswell Park Cancer Institute, Buffalo, NY 14263, USA; 7 University of Colorado Cancer Center, Bldg 500, Suite 6004C, 13001 E 17th Place, Aurora, CO 80045, USA; 8 Young Women’s Breast Cancer Translational Program, University of Colorado Cancer Center, University of Colorado Anschutz Medical Campus, 1665 Aurora Court, Aurora, CO 80045, USA

## Abstract

**Introduction:**

A postpartum diagnosis of breast cancer is an independent predictor of metastases, however the reason is unknown. In rodents, the window of postpartum mammary gland involution promotes tumor progression, suggesting a role for breast involution in the poor prognosis of human postpartum breast cancers. Rodent mammary gland involution is characterized by the programmed elimination of the secretory lobules laid down in preparation for lactation. This tissue involution process involves massive epithelial cell death, stromal remodeling, and immune cell infiltration with similarities to microenvironments present during wound healing and tumor progression. Here, we characterize breast tissue from premenopausal women with known reproductive histories to determine the extent, duration and cellular mechanisms of postpartum lobular involution in women.

**Methods:**

Adjacent normal breast tissues from premenopausal women (n = 183) aged 20 to 45 years, grouped by reproductive categories of nulliparous, pregnant and lactating, and by time since last delivery were evaluated histologically and by special stain for lobular area, lobular type composition, apoptosis and immune cell infiltration using computer assisted quantitative methods.

**Results:**

Human nulliparous glands were composed dominantly of small (approximately 10 acini per lobule) and medium (approximately 35 acini per lobule) sized lobules. With pregnancy and lactation, a >10 fold increase in breast epithelial area was observed compared to nulliparous cases, and lactating glands were dominated by mature lobules (>100 acini per lobule) with secretory morphology. Significant losses in mammary epithelial area and mature lobule phenotypes were observed within 12 months postpartum. By 18 months postpartum, lobular area content and lobule composition were indistinguishable from nulliparous cases, data consistent with postpartum involution facilitating regression of the secretory lobules developed in preparation for lactation. Analyses of apoptosis and immune cell infiltrate confirmed that human postpartum breast involution is characterized by wound healing-like tissue remodeling programs that occur within a narrowed time frame.

**Conclusions:**

Human postpartum breast involution is a dominant tissue-remodeling process that returns the total lobular area of the gland to a level essentially indistinguishable from the nulliparous gland. Further research is warranted to determine whether the normal physiologic process of postpartum involution contributes to the poor prognosis of postpartum breast cancer.

## Introduction

Regardless of age at child birth, women face a transient increase in risk for breast cancer during the postpartum period [1-7]. Clinically, cases diagnosed during or in close proximity to pregnancy have been variably referred to as postpartum or pregnancy-associated [1-6,8,9]. Importantly, a breast cancer diagnosis in the postpartum window confers significantly poorer outcomes, even after adjustments for known clinical prognostic indicators [2,10,11]. These later studies identify a postpartum diagnosis as an independent, poor prognostic factor for young women, and are contrary to some expectations that a diagnosis during pregnancy would have the poorest prognosis [10,12-14]. Recently, using poor prognosis as the criteria for defining postpartum breast cancer, the definition has been expanded to include cases diagnosed within five years of the last childbirth [11,15]. Utilizing this definition, it is estimated that approximately 35% or more of all young women’s cases may be negatively impacted by a recent pregnancy [11,15,16]. It is anticipated that understanding the mechanisms of tumor promotion that occur in the postpartum window will lead to the development of treatment and, possibly, prevention strategies specific to this vulnerable patient population [17].

In rodent models, postpartum mammary gland involution has been identified as a key mediator of tumor progression in the postpartum setting [17,18]. During postpartum gland involution, 80% to 90% of the secretory mammary epithelium developed in preparation for lactation undergoes apoptosis [19-21]. Further, programs similar to wound healing are utilized to remodel the lactation competent rodent gland to a non-secretory state, and include extracellular matrix remodeling, fibrillar collagen deposition, high matrix metalloproteinase activity, and recruitment of macrophages with M2-like attributes [7,18,21-28]. Of note, tissue remodeling involving collagen deposition and macrophage infiltration has demonstrated tumor promotional attributes in multiple models of breast cancer [7,17,25,28-33] and independently predicts poor outcomes in breast cancer patients [28,34,35]. Cumulatively, these studies implicate breast remodeling associated with postpartum involution as a potential mediator of breast cancer progression in young women [8,36].

In pre-clinical models of postpartum breast cancer, non-steroidal anti-inflammatory drugs (NSAIDs) limited in duration to the window of postpartum gland involution reduce tumor growth and metastasis associated with the involution window [17,29]. These studies suggest that an NSAID-based intervention targeted to the physiologic window of postpartum involution may be useful in the treatment or prevention of postpartum breast cancer [15,37]. One critical gap in the knowledge required for rational and safe design of such treatments for women is the lack of a thorough understanding of human postpartum breast involution. Here, using a cohort of premenopausal women with known reproductive histories and undergoing breast biopsy for clinical indications, we characterize postpartum breast involution. We address the extent, duration and cellular mechanisms of the postpartum human breast to determine if lobule regression, epithelial cell death and wound healing-like attributes are observed similar to those described in rodent models.

## Methods

### Study population and reproductive categories

Breast specimens from premenopausal women, 20- to 45-years-old, who underwent clinically indicated biopsies were obtained with Colorado Multiple Institution Review Board approval (COMIRB) under two protocols. One protocol was a retrospective chart review and tissue collection only study deemed exempt from subject consent and Health Insurance Portability and Accountability Act (HIPPA) authorization. The other protocol included informed written patient consent. All cases were de-identified to the research team. One hundred and fifty eight de-identified cases were grouped by reproductive categories of never-been-pregnant (n = 23), pregnant (n = 16), lactating (n = 8), up to 1 month postpartum (n = 6), and postpartum ≤6 months (n = 9), >6 to ≤12 months (n = 5), >12 to ≤18 months (n = 5), >18 to ≤24 months (n = 20), >2 to ≤3 years (n = 7), >3 to ≤6 years (n = 17), >6 to ≤10 years (n = 10) and >10 years (n = 25) since last childbirth. We define never-been-pregnant as cases with no prior evidence of an incomplete pregnancy, which resulted in seven nulliparous cases being excluded from subsequent analysis. The never-been-pregnant cases are referred to as nulliparous hereafter. The final cohort included 151 cases — 120 cancer and 31 benign and included 118 surgical breast specimens and 33 core needle biopsies, which cumulatively are referred to as biopsies herein. Most analyses were performed on subsets of cases (details given below). Self-reported demographic data included 78% Caucasian, 15% Hispanic and 7% women from other races. An independent validation cohort was utilized for lobule analysis, comprised of histological slides from Norwegian women diagnosed with breast cancer ≤40 years of age who were nulliparous (n = 10) or uniparous >10 years postpartum (n = 10).

### Histological staining and imaging

Sections of paraffin embedded tissue (4 μm) were stained with hematoxylin and eosin (H & E) using a Sakura H & E autostainer (Sakura Finetek, Torrance, CA, USA). Stained slides were scanned using an Aperio Scanscope3 system (Aperio, Vista, CA, USA) at 20× corresponding to 0.43 μm per pixel which enables high resolution access to the entire tissue section via a virtual image. Images were evaluated using Imagescope software and Aperio algorithms. Only histologically normal lobules, as determined by a clinically trained pathologist blinded to experimental group [38], were included in analyses. In cases with cancer, normal adjacent lobules, determined by a pathologist, were analyzed a minimum of 5 mm, but on an average of >8 mm, from the cancer.

### Lobular quantification

To quantitate breast architecture, we utilized previously established criteria of binning lobule size into divisions of approximately 1 to 15, >15 to 50, and >50-acini per lobule, also referred to as types 1, 2 and 3, respectively [39,40]. A fourth category, type 4, was identified by a secretory morphology comprised of acini with distended lumen filled with secretions and lined with flattened epithelial cells [39]. Type 4 lobules are considered to be terminally differentiated milk secreting lobules and are the dominant type present in the lactating gland [36]. All morphological analyses were conducted blindly with respect to pathologic diagnosis, reproductive category and patient age. For validation of the lobule type analysis, a total of 70 cases were evaluated with a minimum of 10 normal lobules for each lobule type analyzed per case, except in cases in which fewer than 10 lobules were present where all available lobules were included. For analyses determining if lobular types 1 to 4 are distinguishable by number of nuclei per lobule or by number of nuclei per acinus, nuclear counts were obtained on a subset of cases (n = 30) using JPEG formatted pictures and image J software [41]. For studies where lobular composition of nulliparous cases was evaluated by age, cases were separated into age-groups of ≤30 (n = 4), >30 to ≤35 (n = 6), >35 to ≤40 (n = 9) and >40 to ≤45 years (n = 4), and all lobules present were included in the analyses. To further investigate the effect of age on the baseline breast morphology in this cohort, an expanded analysis of nulliparous and >2 years postpartum cases (≤30 (n = 12), >30 to ≤35 (n = 19), >35 to ≤40 (n = 29) and >40 to ≤45 years (n = 22)) was performed.

To investigate the use of a single surgical specimen as morphologically representative of the whole breast, tissues from each quadrant from three cases were analyzed for total lobular type composition and lobular area. Moreover, to determine if lobular type composition is independent of cancer presence, lobular compositions between cancer (n = 82) and benign (n = 7) cases were compared. For this analysis, we eliminated cases pregnant, lactating and those ≤24 months since the last child birth to minimize the potential influence of pregnancy on gland morphology. The association of lobule type with tumor estrogen receptor (ER) status was determined using ER positive (n = 47) or ER negative (n = 24) cases. To investigate potential influences of menstrual cycle, initial and subsequent breast biopsies from the same woman collected two to three weeks apart were compared for lobular type composition (n = 12).

A detailed analysis of percent lobular area was done on a subset of cases; nulliparous (n = 12), pregnant (n = 15), lactation (n = 8) and postpartum cases based on time since last child birth (n = 58). Further, all 151 cases in the young women’s cohort were analyzed for lobular type composition. Lobular type composition for individual cases was then compiled and compared by reproductive categories. A corroborative analysis for comparison of lobular composition in nulliparous and uniparous >10 years postpartum cases was performed on an independent sample from Norway (n = 20). Also, detailed analysis comparing lobule number, size and acini number per lobule was done for nulliparous (n = 12 cases) and >2 years parous (n = 17 cases) groups.

### Immunohistochemistry and special staining

Breast tissue slides were pretreated with Dako TRIS or EDTA antigen retrieval solution (Dako North America Inc., Carpinteria, CA, USA) at 120 °F under pressure for five minutes. Ki-67 was used to identify cells undergoing proliferation (1:400, Dako M7240). TACS 2TdT Fluorescein apoptotic kit (Trevigen Inc., # 4812-30-K, Gaithersburg, MD, USA) was implemented to visualize apoptotic cell death. CD45, a common leucocyte marker which is present on the surface of nucleated hematopoietic cells, was used for identifying all immune cells (1:200, Dako M0701, one hour incubation) [42]. CD68, a macrophage lysosome-associated protein [43], was used to identify macrophages (1:300, Dako # M0876, one hour incubation). Immunoreactivity was detected using Envision + system (Dako) with 3, 3′-diaminobenzidine used as the chromogen for all stains.

### Apoptotic and immune cell quantification

To quantify apoptosis, actively involuting cases at 10 days postpartum in the absence of lactation (n = 1), and weeks one (n = 2), two (n = 1), four (n = 1) and six (n = 1) post-lactation were stained for terminal deoxynucleotidyl transferase dUTP nick end labeling (TUNEL) positive apoptotic cells. Ten lobules with lactational and ten lobules with involutional morphology per case were analyzed and the signal quantified with Image J software. Quantification for CD45 and CD68 was performed in 10 lobules per lobular type per case utilizing a subset of 97 and 77 cases, respectively. Modified de-convoluted Aperio algorithms were implemented for signal quantification.

### Statistical analysis

Mean and standard error were used to summarize expression levels for lobular area, lobular type composition, apoptosis and immune cell infiltration. Analysis of variance (ANOVA) was used to compare mean values between reproductive categories (nulliparous, pregnant, lactation, ≤1 month, >1 to ≤6, >6 to ≤12, >12 to ≤18, >18 to ≤24 months, ≥2 to ≤6 years, >6 to ≤10 years and >10 years postpartum. If the overall F test was significant, comparisons between categories were carried out. If multiple samples were taken from the same patient, correlation among the samples was taken into account during the analysis (random effect ANOVA). Two-group t-test was used instead of ANOVA if there were only two categories in the analysis. *P*-values ≤0.05 were considered statistically significant throughout the paper. SAS software 9.2 (SAS Inc. Cary, NC, USA) was used for all analyses.

## Results

### Method comparisons for lobule type quantification and baseline breast composition

**Figure 1:**
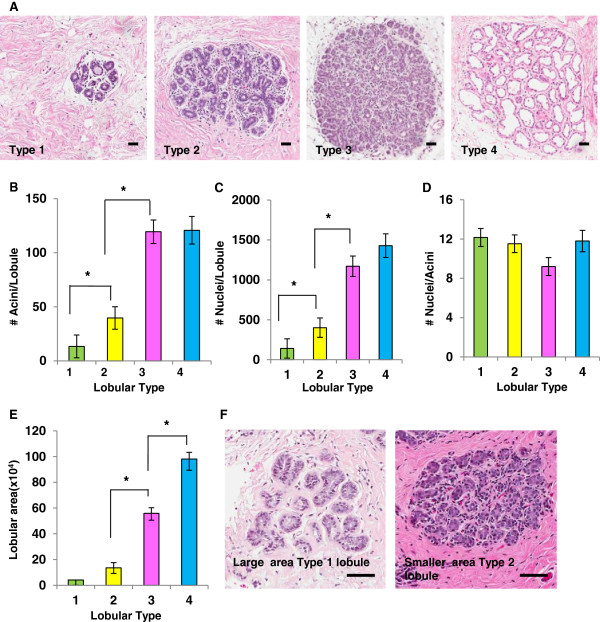
**Criteria for distinguishing between lobular types. A**. H & E stained images of lobules representative of types 1 to 4 (Scale bar = 30 μm). **B**. Validation of acini count per lobule to distinguish lobule types, identifying type 1 (green) as approximately 10 ± 4 acini per lobule, type 2 (yellow) as approximately 35 ± 5 acini per lobule, type 3 (pink) as approximately 114 ± 10 acini per lobule and type 4 (blue) based on secretory morphology with differences between types 1, 2 and 3 (*P ≤0.0007), but not types 3 and 4 (*P* = 0.92) (n= >1,700 lobules). **C**. Number of nuclei per lobule quantitated for lobular types 1, 2 and 3 (*P ≤0.002) and types 3 and 4 (*P* = 0.23) (n= >230 lobules). **D**. Number of nuclei per acini evaluated for each lobular type shows no statistical difference (*P* ≥0.07) (n= > 230 lobules). **E**. Lobular area distinguishes between lobules types 2, 3 and 4 (*P ≤0.0001) but not between types 1 and 2 (*P* = 0.11). **F**. H & E stained sections showing lobule types 1 and 2 occupying approximately the same area (approximately 50,000 μm^2^) but categorized as different types based on the number of acini/lobule (Scale bar = 90 μm). All error bars represent standard error of the mean (SEM).

The breast lobular component expands and differentiates with pregnancy and lactation transforming the relatively rudimentary gland into an alveolar rich lactation-competent structure [44]. Specifically, the human breast has been reported to consist predominantly of small type 1 and 2 lobules before pregnancy, large type 3 lobules during pregnancy and mature secretory type 4 lobules during lactation [39]. A requisite to the study of the extent and duration of postpartum breast involution is the validation of morphometric methods used to characterize breast lobular types. In our cohort of 151 premenopausal women, 20- to 45- years-old (Additional file 1: Table S1 and Additional file 2: Table S2), wide variation in breast lobule size between cases was evident (Figure 1A). To validate that acini number reproducibly distinguishes between these observed lobule types, 70 randomly selected cases representing all reproductive categories were scored for acini per lobule, for a total analysis of >1,700 individual lobules. In these cases, lobule type 1 had an average 10 ± 4 acini per lobule, type 2 had an average of 35 ± 5 acini per lobule and type 3 had an average of 114 ± 10 acini per lobule, with type 4 defined by a secretory phenotype (Table 1). We found that the number of acini per lobule distinguished between lobule types 1, 2, and 3 (*P* <0.007), but did not distinguish between type 3 and 4 (*P* = 0.92) (Table 1 and Figure 1B). Classification of lobular types based on mean nuclear count similarly demonstrated that lobule types 1, 2 and 3 were distinguishable (*P* ≤0.0002), but types 3 and 4 were indistinguishable (*P* = 0.23) (Figure 1C). These nuclear count data also indicated that lobule maturation of type 3 to type 4 does not involve a significant increase in epithelial cell proliferation. However, evidence for direct pathways between lobular types cannot be directly discerned from morphological analysis. Further, the number of nuclei per acinus remained constant irrespective of lobular type (Figure 1D), suggesting that the acinus is the unit of lobular expansion in the human breast.

**Table 1:** Validation of acinar count method for lobule typing

**Lobule type**	**Criteria for lobule type based on number of acini/lobule **[39]	**Number of cases analyzed/lobule type**	**Total number of lobules analyzed**	**Mean number of lobules analyzed/case (± SD)**	**Observed number of acini/lobule**
Type 1	12	63	759	12.6 ± 11	10 ± 4
Type 2	50	53	482	9.1 ± 8.4	35 ± 5
Type 3	80	36	226	6.3 ± 4.8	114 ± 10
Type 4	Secretory morphology	13	150	11.5 ± 9.8	128 ± 27

The average number of acini per lobule per type in the breast tissue samples evaluated (last column) are not different than published acinar numbers previously observed in human breast tissue (second column, reference 39), validating the acinar count method for subsequent analyses.

Measuring lobular-area is an alternative method to assess lobule differentiation state. As expected, the lobular area increased with lobular complexity, and types 2, 3 and 4 were readily identified using this method (*P* <0.0001) (Figure 1E). The ability of this area-method to distinguish between types 3 and 4 reflects the increase in type 4 size due to milk production and secretion (Figure 1A). However, using this area-method, type 1 and 2 lobules did not differ significantly (*P* = 0.11) (Figure 1E), which may be due to a wide variation in stromal to epithelial ratios within these smaller lobules (Figure 1F and Additional file 3: Figure S1). Since area-analysis does not distinguish between lobule types 1 and 2, for subsequent analyses, we utilized number of acini per lobule to distinguish between types 1, 2 and 3, and lactational morphology to identify type 4.

**Figure 2:**
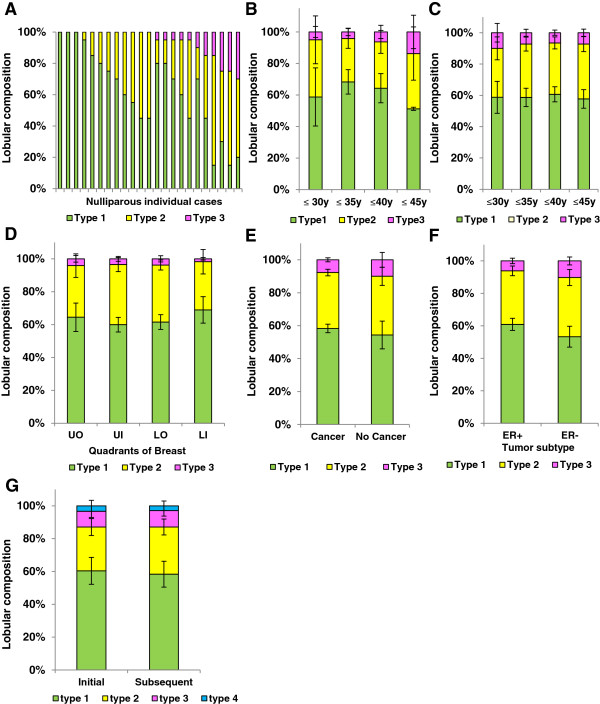
**Heterogeneity in lobular composition is independent of age, breast quadrant, and presence of cancer. A**. Individual lobular composition in a cohort of nulliparous women ≤45 years of age (n = 23 cases). Among women, there is marked variation in lobule type; type 1 (green), type 2 (yellow) and type 3 (pink) (n= >550 lobules). **B**. Lobular composition of breast in nulliparous women does not vary by age increments of five years (≤35 years versus ≤30 years (*P* = 0.89); ≤40 years versus ≤30 years (*P* = 0.62); ≤45 years versus ≤30 years (*P* = 0.19)) **C**. Lobular composition in an expanded cohort excluding recently pregnant and lactating women (n = 82 cases) similarly shows lobular composition is not age dependent (≤35 years versus ≤30 years (*P* = 0.35); ≤40 years versus ≤30 years (*P* = 0.18); ≤45 years versus ≤30 years (*P* = 0.34)) **D**. Breast quadrant analysis from three mastectomy cases demonstrated no differences in lobular types for all quadrants (LO versus LI (*P* = 0.33); UI versus LI (*P* = 0.40); UO versus LI (*P* = 0.25)). **E**. Lobular type composition does not vary between women who were subsequently diagnosed with cancer (n = 82 cases) or were cancer free (n = 7 cases) (*P* = 0.59), or **(F)** by the presence (n = 47 cases) or absence (n = 24 cases) of ER expression in the adjacent tumor (*P* = 0.15). **G**. Lobular type composition is not significantly influenced by hormonal effects of the menstrual cycle as depicted by initial and subsequent breast biopsies two to three weeks apart (n = 12 cases) for type 1 (*P* = 0.38), 2 (*P* = 0.32) and 3 (*P* = 0.46) lobules. All error bars represent standard error of the mean (SEM). LI, lower inner; LO, lower outer; UI, upper inner; UO, upper outer.

We next investigated variation in lobule architecture in the nulliparous group. Among our nulliparous patients (n = 23), we evaluated >550 lobules and the dominant lobule types were the smaller types 1 and 2; however, significant inter-case variation was evident. Among nulliparous women, lobule composition ranged from 100% to 20% type 1, 60% to 0% type 2, and 30% to 0% type 3 (Figure 2A). Surprisingly, some type 3 lobules were present in 48% of these nulliparous cases, demonstrating that a prior complete or incomplete pregnancy does not appear to be required for their development (Figure 2A). Terminally differentiated, type 4 lobules were not found in these nulliparous cases (Figure 2A). Overall, this analysis establishes a baseline variation in lobule composition, data required to investigate effects of pregnancy, lactation and postpartum involution on breast lobule architecture in our young women’s breast tissue cohort.

### Effect of potential technical confounders in assessing breast architecture

To utilize lobular composition to address questions of extent, duration and individual variation in postpartum breast involution, it is necessary to investigate the influence of clinical and technical parameters which may confound lobule-type quantitation. Here, we address patient age, use of a single tissue section per case, cancer diagnosis, biologic cancer type and potential impact of menstrual cycle on lobular type composition. In the nulliparous group (n = 23 cases), patient age was not found to significantly impact lobular type composition (*P* ≥0.19) (Figure 2B), nor was age found to impact lobule type composition in an extended cohort (n = 82 cases) consisting of combined nulliparous and parous cases >2 years postpartum (*P* ≥0.18) (Figure 2C). We next addressed the use of a single tissue specimen per case as a potential methodological limitation by performing quadrant analyses on three parous cases >5 years postpartum. As expected, variation in lobular composition between individuals was observed (Additional file 4: Figure S2a). Further, within the same subject, the largest mean difference in lobule type composition was found between the lower inner and upper outer quadrants (Additional file 4: Figure S2a). However, a mixed effect model, which takes the correlation among the tissue samples from the same case into account, indicates that quadrant location is not significantly associated with the presence of lobular types (lower outer (LO) versus lower inner (LI) (*P* = 0.33); upper inner (UI) versus lower inner (LI) (*P* = 0.40); upper outer (UO) versus lower inner (LI) (*P* = 0.25)]. Further, an aggregate analysis revealed no significant morphological differences between quadrants in these cases (*P* ≥0.25) (Figure 2D). To address the potential confounder of malignancy, we compared adjacent normal lobular composition of women diagnosed with benign breast lesions to those with cancer. We found the groups indistinguishable (*P* = 0.59), indicating that in normal adjacent tissue at least 5 mm distant from the breast cancer, the presence of cancer does not significantly alter lobule type composition (Figure 2E). Next, we examined whether ER expressivity of the breast tumor might influence lobule composition, as variation in lobule types has been reported between patients with luminal A and basal breast cancers [38]. In our cohort, differences in lobule type composition were not observed between ER positive (n = 47) and ER negative (n = 24) cases (*P* = 0.15) (Figure 2F). Finally, to address the potential effect of menstrual cycling, lobule composition for types 1, 2, 3 and 4 lobules was analyzed in initial and subsequent biopsy tissues collected two to three weeks apart in a subset of premenopausal women (n = 12). Although there were small variations in lobular composition between biopsy times, no significant differences between type 1 (*P* = 0.38), type 2 (*P* = 0.32) or type 3 (*P* = 0.46) lobules were observed (Figure 2G). To summarize, in this premenopausal cohort, lobule type composition varies more between women of the same reproductive category than between categories defined by patient age, biopsied quadrant, ER expression of the tumor, presence of breast cancer and stage of menstrual cycle. Although, overall, the power of statistical tests is low due to low sample size, the similarities in mean values indicate that these specific parameters have no strong influence on lobule type analysis in the postpartum involution window, which is the primary objective of this study.

### Evidence for postpartum involution

**Figure 3:**
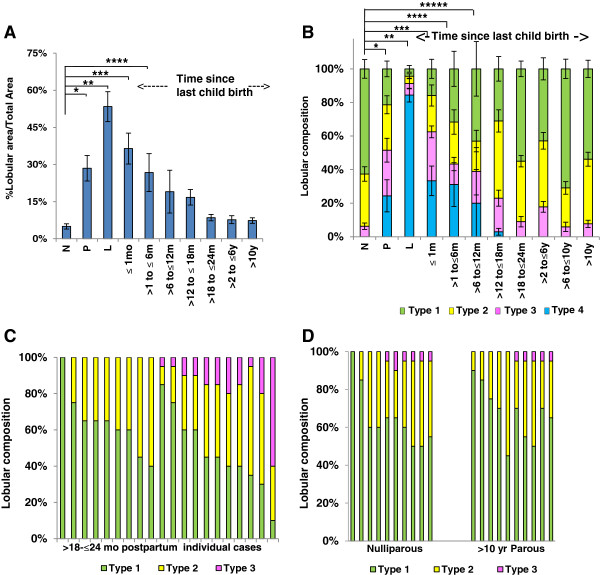
**Histological evidence of postpartum involution in human breast. A**. Percent lobular area by reproductive groups of nulliparous (N) (n = 12), pregnant (P) (n = 15), lactation (L) (n = 8), up to 1 (n = 6), >1 to ≤6 (n = 9), >6 to ≤12 (n = 5), >12 to ≤18 (n = 5), >18 to ≤24 months (n = 12), >2 to ≤6 years (n = 11) and >10 years (n = 10) since last child birth. Percent lobular area is significantly different from N during P (*P <0.0001), L (**P <0.0001), ≤1 (***P <0.0001), and at >1 to ≤6 months (****P = 0.0007) postpartum, and not statistically different at 12 months postpartum (*P* = 0.07). **B**. Breast lobular composition across the reproductive groups of N (n = 23), P (n = 16), L (n = 8), up to 1 (n = 6), >1 to ≤6 (n = 9), >6 to ≤12 (n = 5), >12 to ≤18 (n = 5), >18 to ≤24 months (n = 20), and >2 to ≤6 (n = 24), >6 to ≤10 (n = 10) and >10 years (n = 25) since last child birth. The lobular type composition is different from the N group during P (*P <0.001), L (**P <0.001), ≤1 month (***P <0.0001), >1 to ≤6 months (****P = 0.0001), >6 to ≤12 months (*****P = 0.001) postpartum, and is indistinguishable by 18 months postpartum (*P* = 0.08). **C**. Lobular composition of individual women who are >18 to ≤24 months postpartum (n = 20). **D**. An independent Norwegian cohort comprising Nulliparous (n = 10) and >10 years parous (n = 10) cases shows no lobular differences between groups. Type 1 (*P* = 0.72), 2 (*P* = 0.81) and 3 (*P* = 0.47). All error bars represent standard error of the mean (SEM).

To determine if breast tissue developed for lactation involutes during the postpartum period in women, as observed in rodent models [7,24], we assessed lobular area and lobular composition across nulliparous, pregnant, lactating and postpartum groups defined by time since last childbirth (n = 93). Lobular area in breast tissue of pregnant women (mean = 26.84, 95% confidence interval (CI) (15.7, 37.9)) showed a five-fold increase compared to nulliparous women (mean = 5.00, 95%CI (-3.2, 13.2)) (*P* <0.0001) (Figure 3A). An additional two-fold increase (mean = 48.4, 95%CI (35.3, 61.4)) in lobule area occurred with lactation (*P* <0.0001) (Figure 3A). However, by 18 months postpartum the lobular area of the gland was indistinguishable from the pattern observed in nulliparous women (*P* = 0.25). It is well documented that in addition to an increase in lobular area, there is increased lobular complexity with pregnancy and lactation [39,45-47] and, as expected, with pregnancy we found the lobular type composition to be highly shifted to type 3 and type 4 differentiated lobules, with evidence for further maturation to type 4 lobules with lactation (n = 151) (Figure 3B and Additional file 4: Figure S2b). As with lobular area, by 18 months postpartum, lobular type composition of the parous group was indistinguishable from the nulliparous group (*P* ≥0.65) (Figure 3B). These data indicate complete loss of the type 3 and type 4 lobules developed for lactation within a relatively narrow window of time. A sub-analysis of lobular type composition in women who were 18 to 24 months postpartum confirmed high similarity with our nulliparous cohort (compare Figure 3C with Figure 2A). In an independent cohort of breast tissue obtained from premenopausal women from Norway, on comparing nulliparous and >10 years postpartum categories, we found no significant differences in lobule type composition (type 1 (*P* = 0.72), 2 (*P* = 0.81) and 3 (*P* = 0.47)) (Figure 3D), providing additional support for postpartum involution of the lobules expanded during pregnancy and lactation. Combined, these morphological data strongly suggest that the secretory mammary epithelium developed in preparation for lactation undergoes involution within 12 to 18 months of cessation of lactation, and further, the regressed glandular phenotype is maintained in the absence of subsequent pregnancy. Further, analysis of >10 year postpartum cases (n = 25) grouped by uniparous and multiparous status showed no correlations between the presence of lobular type 3 and the number of completed pregnancies (Additional file 5: Figure S3), suggesting loss of differentiated lobules following each pregnancy.

**Figure 4:**
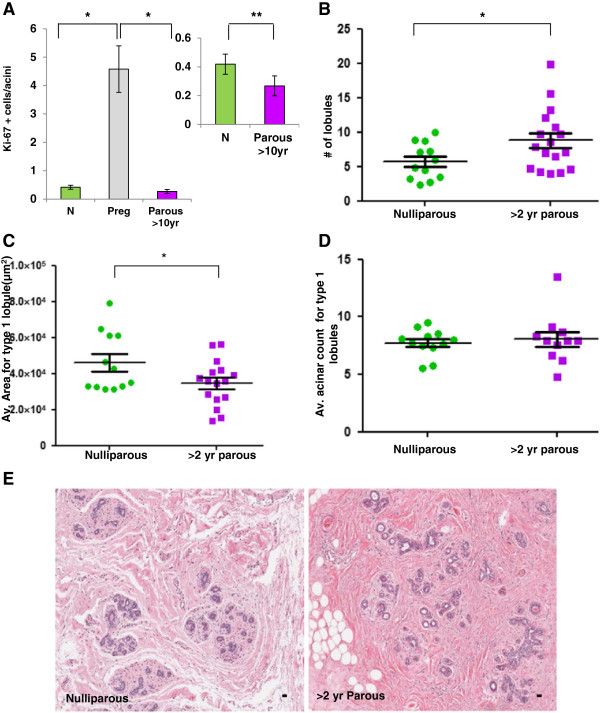
**Lobular differences in nulliparous and parous breast tissue. A**. Significant differences in proliferative index using Ki-67 between breast lobules of nulliparous (N) (green), pregnant (Preg) (gray) and parous >10 year (purple) women. Lobules of Nulliparous and parous >10 years are different from Preg lobules (*P <0.0001). The inset shows there is a significant decrease in proliferative capacity in parous breast lobules when compared to Nulliparous breast lobules (**P = 0.030). **B**. Lobule count data show an increase in number of lobules per unit area in >2 years postpartum when compared to the Nulliparous breast tissue (*P = 0.04). **C**. The average area of type 1 lobules is significantly increased in Nulliparous breast tissue compared to type 1 lobules in >2 years postpartum breast issue (*P = 0.04). **D**. No significant differences were noted on comparing the acinar count of type 1 lobules between the Nulliparous and >2 years postpartum breast tissue (*P* = 0.66). **E**. The H & E stained images show Nulliparous breast tissue with a reduced number of lobules per area and larger size type 1 lobules when compared to >2 years postpartum breast tissue (Scale bar = 30 μm). All error bars represent standard error of the mean (SEM).

As previously reported, we also observed an increase in proliferative capacity with pregnancy and confirmed previous reports of decreased proliferative capacity in lobules of parous compared to nulliparous lobules (Figure 4A) [39]. Subsequent morphometric analysis demonstrated an increase in number of lobules per unit area in the parous compared to the nulliparous gland (*P* = 0.04) (Figure 4B). The observation that lobule number increases with parity but overall area of the gland composed of lobules does not (compare Figure 4B with Figure 3A) suggests that the fully regressed lobules of the parous gland are smaller than in the nulliparous gland. A sub analysis of type 1 lobules confirmed reduced lobule size in parous compared to nulliparous glands (Figure 4C), which is not accounted for by a decrease in acini number per lobule (Figure 4D). Similar trends were observed for type 3, but not type 2 lobules (Additional file 6: Figure S4a,b). Representative H & E images of type 1 lobules in the nulliparous gland depict reduced lobule number and increased lobule size in comparison to a >24 month postpartum case (Figure 4E). In total, these data demonstrate similarity in lobule area and lobule type in the nulliparous and parous cases >24 months postpartum, consistent with postpartum involution, but also demonstrate decreased proliferative capacity, increased number and decreased lobule size with parity.

### Histological hallmarks of postpartum involution

**Figure 5:**
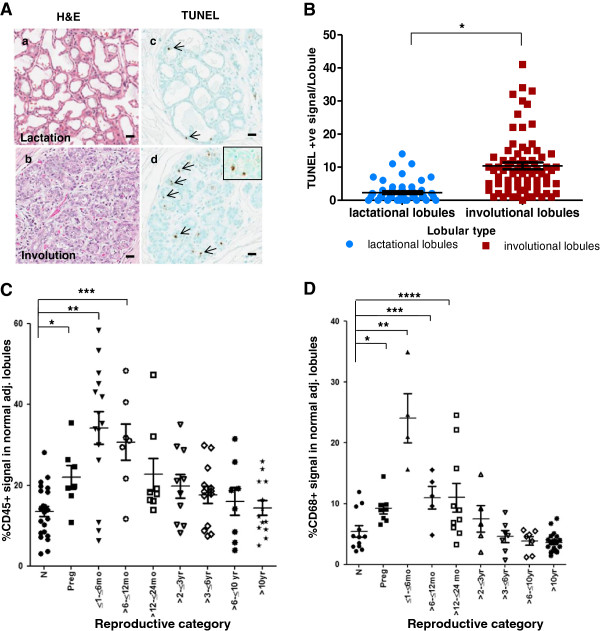
**Evidence for apoptosis and immune cell infiltration during postpartum breast involution. Aa**, **b**. H & E stained sections showing lactational and involutional morphology of lobules within an actively involuting postpartum breast. **Ac**, **d**. Apoptosis is evident in lobules with involutional morphology as detected by TUNEL stain (TdT nick end labeling) (arrow). Inset shows single acini in an involutional lobule with two apoptotic epithelial cells. **B**. Quantitation of TUNEL staining in lactational and involutional lobules from early involution breast tissue obtained from six women (*P <0.0001). **C**. Quantification of CD45^+^ immune cell numbers across reproductive groups of nulliparous (N) (n = 24), pregnant (P) (n = 7), ≤1 to ≤6 (n = 9), >6 to ≤12 (n = 7) and >12 to ≤24 months (n = 6), and >2 to ≤3 (n = 10), >3 to ≤6 (n = 13), >6 to ≤10 (n = 8) and >10 years (n = 13) postpartum. When compared to nulliparous cases there is a significant increase in CD45^+^ cells during pregnancy (*P = 0.005), ≤1 to ≤6 months (**P = 0.0002) and up to >6 to ≤12 months postpartum (***P = 0.01). **D**. Quantification of CD68^+^ macrophages by reproductive groups including nulliparous (N) (n = 12), pregnant (P) (n = 8), and ≤6 (n = 4), >6 to ≤12 (n = 5) and >12 to ≤24 months (n = 10), and >2 to ≤3 (n = 5), >3 to ≤6 (n = 7), >6 to ≤10 (n = 7) and >10 years (n = 19) postpartum. There is a significant increase in CD68^+^ cells during pregnancy (*P = 0.01), ≤1 to ≤6 (**P = 0.0001), >6 to ≤12 (***P = 0.01) and up to 24 months postpartum (****P = 0.02) when compared to nulliparous cases. All error bars represent standard error of the mean (SEM).

We next investigated whether postpartum breast involution in women is characterized by increased alveolar cell apoptosis, a well-documented hallmark of postpartum mammary gland involution in rodents [7,18,19,23,24]. To assess for apoptosis in human involuting breast, we identified six cases within six weeks post-weaning, as alveolar cell apoptosis is an early involution event in rodents [21,48]. Breast tissue from these cases were comprised of lactational, type 4 lobules as well as condensed, non-secretory, type 3 lobules, morphologies consistent with ongoing involution (Figure 5Aa,b). In these cases, apoptotic cells, as assessed by immunohistochemistry (IHC) detection of DNA fragmentation, were elevated five-fold in condensed lobules compared to lobules with a lactational phenotype (*P* <0.0001) (Figure 5Ac,d, 5B). Furthermore, many individual acini contained multiple TUNEL-positive cells (Figure 5Ad, inset). These data are consistent with programmed cell death of the secretory epithelium contributing, at least in part, to the dramatic decrease in glandular tissue observed during postpartum breast involution.

Recently, it has been observed that postpartum mammary gland involution is characterized by an influx of immune cells thought to be integral to tumor promotion [7,25,28,30]. For example, using human breast tissue composed of both lactational and actively involuting lobules, we previously demonstrated high levels of CD45+ leukocytes and CD68+ macrophages in lobules with an involuting morphology [7]. However, these studies have not identified the duration of immune cell infiltrate during postpartum breast involution, which is of potential relevance since immune infiltrate may contribute to the poor prognosis of postpartum breast cancer [29]. To assess the duration of immune cell infiltrate, we investigated lobular content of CD45+ and CD68+ cells by defined times postpartum. We found that CD45+ cells were maximally elevated within six months postpartum and remained significantly elevated up to twelve months postpartum compared to the nulliparous group (*P* = 0.01) (n = 97) (Figure 5C). Evidence for a further and gradual decline in CD45 for up to 10 years postpartum is also observed; however, a larger cohort is required to determine more precisely the pattern of CD45+ cell efflux in the postpartum breast. CD68+ levels were also maximal in the ≤6 months postpartum group (Figure 5D), and remained elevated through ≤24 months postpartum (*P* = 0.02) (n = 77) (Figure 5D), returning to nulliparous levels by 3 years postpartum. Dual staining revealed that approximately 60% of CD45+ cells were CD68+ as well (data not shown). These data indicate that leukocytes, in general, and macrophages, specifically, persist in the postpartum gland beyond the 12 to 18 month time frame when morphological evidence shows complete lobule regression.

## Discussion

Wound healing and inflammatory milieus are strongly implicated in breast cancer incidence and progression [25,28,30,34,49]. In rodents, physiologically normal postpartum mammary gland involution has wound healing and immunomodulatory properties and promotes breast cancer growth, invasion and metastasis in xenograft models [7,17,25,28,29]. Similar wound healing and immunologic attributes in the human breast may help explain the poor prognosis of breast cancers diagnosed within five years of a completed pregnancy [7,18,25,28,30,36,49,50]. Here, we confirm conservation of tissue mechanisms of postpartum mammary gland involution between rodents and humans, including massive epithelial cell loss and acquisition of immune attributes previously correlated with pro-tumorigenic microenvironments [7,28,30]. These studies begin to identify a narrowed time period in a postpartum woman’s life that may represent a unique window of increased breast cancer risk and progression.

In rodents, postpartum mammary gland involution is primarily characterized by programmed apoptotic death of the secretory mammary epithelium [19,51]. In our premenopausal human cohort, a complete loss of the differentiated lobules developed in preparation for lactation is observed by 18 months postpartum. This reversal of lobular area and type composition to baseline levels is consistent with epithelial cell death being the primary cellular mechanism driving postpartum involution. High levels of mammary epithelial cell apoptosis observed in breasts within six weeks post-weaning corroborate this hypothesis. Further, CD45+ leukocyte and CD68+ macrophage infiltration into the involuting human breast suggests the use of wound healing-like programs to remodel the pregnant/lactating gland to its non-secretory state. Given the evidence for immune cell promotion of breast cancer [28,31,50,52], this immune infiltrate into the involuting breast may contribute to the poor prognosis of women diagnosed postpartum.

Previously published data have shown persistence of differentiated type 3 lobules in breasts of premenopausal parous women [39,45-47]. Our data showing no differences in total lobule area and type composition between nulliparous and parous cohorts are contrary to these published data. Based on the small differences we observed in type 3 lobule composition between nulliparous and parous cohorts, we calculate that approximately 775 premenopausal breast biopsies are required to adequately address the question of whether type 3 lobules persist in parous women. Further, our analysis is limited in that we have not performed gene expression studies, which have demonstrated persistent gene expression changes consistent with lobule differentiation with parity [45,53,54]. Consistent with previous reports [39], we did observe a decrease in the proliferative capacity of mammary epithelial cells with completed pregnancy as well as an increase in the total number of lobules present. Additional clinical variables that may contribute to why persistent type 3 lobules were observed in previous studies but not in ours include differences of ethnicity, basal metabolic index, hormone based contraceptive use, and/or familial history of breast cancer.

A previous study comprising 9,000 cases encompassing all ages reported age-related breast involution in approximately 8.5% of women less than 40-years-old [40]. In our premenopausal cohort, we did not identify age-related involution. Several factors, including the narrower age range investigated in our study, the exclusion of postmenopausal cases, the relatively small size of our cohort and differences in morphometric endpoints utilized to measure lobular involution likely contribute to differences observed between studies.

Our study design has several strengths including the use of never-been-pregnant cases for our nulliparous group, permitting for the first time an evaluation of baseline breast morphology in the absence of a prior pregnancy, including incomplete pregnancies. Our morphometric analysis is also an asset, as the semi-quantitative IHC approach permits computer generated data collection within the entire tissue section and acquisition of continuous rather than categorical data, which reduces the potential for inadvertent operator bias. Further, our study sample size is the largest cohort to date to assess postpartum breast involution based on verification of clinical medical records for date of last pregnancy. Another important strength of our study is that we have limited our analysis to premenopausal women. Other studies investigating normal mammary gland morphology have encompassed broader age ranges and pre- and post-menopausal status, making it more difficult to discern between postpartum, age-related and menopause-induced breast involution [40,55].

A potential limitation of our study is the availability of a single tissue specimen per case as a representative of the whole breast specimen. To address this limitation, we performed lobular composition analysis for three mastectomy specimens and found that a single biopsy can be globally representative of the entire breast tissue. While our results confirm previous reports demonstrating some variation in lobule complexity between breast quadrants [39], our data also support the work of others indicating that the use of a single tissue analysis is relatively representative of global tissue architecture [40,55].

Another limitation of our study is the low number of cases consisting of true normal breast tissue in the absence of any pathological lesion. Recently, true normal, reduction mammoplasty and benign-disease breast tissues were found to differ in the process of age-related involution, with true normal breast showing increased regression [56]. Here, we observed similar trends in magnitude and duration of postpartum breast involution between cancer and benign cases, suggesting that postpartum breast involution is a dominant regression program that occurs relatively independent of adjacent pathology. However, repeating a postpartum breast involution study in true normal tissue remains an important objective. Lastly, our cohort lacks patient ethnic diversity and is enriched for older age mothers with low gravidity. Published studies show that Hispanic and African American women have younger age at first pregnancy, higher gravidity, distinct nursing histories [57-59], and experience a higher incidence of young women’s breast cancer than Whites [59-61]. In order to further investigate the feasibility of developing prevention and treatment strategies targeted to the postpartum window, it will be essential to determine if postpartum breast involution is modified by race, reproductive and lactation histories, as well as risk profiles.

## Conclusions

This study identifies postpartum breast involution in women as a dominant window of tissue remodeling characterized by massive loss of differentiated lobules, apoptotic epithelial cell death and immune infiltrate associated with breast cancer progression in preclinical models. Further research into the mechanisms of human postpartum breast involution and its potential tumor promoting attributes may lead to novel prevention and treatment strategies for postpartum breast cancer. A potential asset would be the limited duration of treatment required to target this narrowed window of risk.

## Abbreviations

ANOVA: analysis of variance; ER: estrogen receptor; H & E: hematoxylin and eosin; IHC: immunohistochemistry; L: lactation; LI: lower inner; LO: lower outer; N: nulliparous; NSAID: non-steroidal anti-inflammatory drugs; P: pregnant; TUNEL: terminal deoxynucleotidyl transferase dUTP nick end labeling; UI: upper inner; UO: upper outer.

## Competing interests

The authors declare that they have no competing interests.

## Authors’ contributions

SJ conducted the pathology review of the histopathology slides, collected and analyzed the data and co-wrote the manuscript. DG conducted the statistical analysis for the study and co-wrote the manuscript. PB conducted the embedding, sectioning and staining of the human cases and revised the manuscript. GA contributed with all necessary approvals and random selection of breast tissue blocks from Norway and critically revised the manuscript. SME, CBA and ADT conducted extensive chart review and selected human cases for this study and critically revised the manuscript. VFB contributed to obtaining grant funding, contributed to obtaining all necessary approvals and clearances to conduct the research, supervised the analysis of data and co-wrote the manuscript. PS conceived and designed the study, obtained grant funding, supervised the research, analyzed the data and principally wrote the manuscript. All the authors read and approved the final manuscript.

## Supplementary Material

Additional file 1: Table S1 Clinical characteristics of cases included in the study. Click here for file

Additional file 2: Table S2 Tumor characteristics of cases included in the study. Click here for file

Additional file 3: Figure S1 Area-analysis cannot distinguish between lobular types 1 and 2. Some type 1 lobules can occupy larger areas when compared to smaller type 2 lobules due to variation in stromal to epithelial ratio. Eighteen type 1 lobules and 13 type 2 lobules were included in this analysis. All error bars represent standard error of the mean (SEM). Click here for file

Additional file 4: Figure S2 Lobular composition by breast quadrant and cancer free cases. **S2a**: Lobular composition in individual breast quadrants obtained from bilateral mastectomy specimen of three women who were six to eight years postpartum. Each individual graph represents morphological variation in lobular composition for upper outer (UO), upper inner (UI), lower outer (LO) and lower inner (LI) quadrants of left (L) and right (R) breasts for each woman. **S2b**: Lobular composition across the reproductive cycle in women diagnosed with benign breast pathology. This cancer-free cohort of nulliparous (N) (n = 5), pregnant (P) (n = 11), lactation (L) (n = 8), >1 to ≤6 months (n = 5), >2 to ≤6 years (n = 1), and >9 years (n = 1) postpartum cases shows similar lobular composition as that of the total cohort (Figure 3B), suggesting that at the morphological level, postpartum involution is not significantly affected by the presence of adjacent tumor. All error bars represent standard error of the mean (SEM). Click here for file

Additional file 5: Figure S3 Percent area composed of type 1, 2 and 3 lobules does not change with parity status. Percent of tissue composed of type 1, 2 and 3 lobules shows no correlation with the number of completed pregnancies, suggesting similar gland regression with each round of postpartum involution. Click here for file

Additional file 6: Figure S4 Type 2 and 3 lobule size does not vary between nulliparous and parous cases. **S4a**. No differences are noted in average area of type 2 lobules between >2 years parous and nulliparous cases. **S4b**. The average area of type 3 lobules shows a trend towards the area being decreased in parous breast tissue compared to nulliparous breast issue (*P* = 0.06). All error bars represent standard error of the mean (SEM). Click here for file
