# Supplementary material for: Postpartum breast involution reveals regression of secretory lobules mediated by tissue-remodeling
Source: Breast Cancer Res. 2014 Mar 28;16(2):R31. doi: 10.1186/bcr3633 (PMC4053254; doi:10.1186/bcr3633)
Supplement: Additional file 1: Table S1 — Clinical characteristics of cases included in the study. [file bcr3633-S1.pdf]

Supplementary Table 1S

Clinical characteristics of cases included in the study

Reproductive categories	*Number of cases*	*Av. Age*	*Av. Gravidity*	*Av. Parity*	*% of cases with breast cancer*
Nulliparous	23	35.0	0	0	78.2
Pregnant	16	31.2	2	1	31.3
Lactation	8	36	2	2	0
Up to 1mo	6	33.8	2	2	100
>1- ≤ 6m	9	32.3	2	2	44.5
>6- ≤ 12m	5	28.0	3	2	100
>12- ≤ 18m	5	37.0	3	2	100
>18- ≤ 24m	20	36.7	2	2	95
>2- ≤ 3y	7	34.1	3	2	100
>3- ≤ 6y	17	35.5	3	2	100
>6- ≤ 10y	10	39.2	3	3	100
>10y	25	39.7	5	2	96
