# Supplementary material for: Postpartum breast involution reveals regression of secretory lobules mediated by tissue-remodeling
Source: Breast Cancer Res. 2014 Mar 28;16(2):R31. doi: 10.1186/bcr3633 (PMC4053254; doi:10.1186/bcr3633)
Supplement: Additional file 2: Table S2 — Tumor characteristics of cases included in the study. [file bcr3633-S2.pdf]

Supplementary Table 2S

Tumor characteristics of cases included in the study

Parity	***N***	***P***	***L***	***≤1 mo***	***>1≤6 mo***	***>6≤12 mo***	***>12≤18 mo***	***>18≤24 mo***	***>2≤3 yr***	***>3≤6 yr***	***>6≤10 yr***	***>10 yr***
Cancer	18	5	0	6	4	5	5	19	7	17	10	24
Benign	5	11	8	0	5	0	0	1	0	0	0	1
Histologic Subtype												
Ductal(D)	15	5	0	6	4	4	4	18	6	13	10	20
Lobular(L)	1	0	0	0	0	1	0	0	1	2	0	1
D+L	2	0	0	0	0	0	0	0	0	0	0	0
other	0	0	0	0	0	0	1	0	0	2	0	3
Biological Subtype												
Luminal A	7	1	0	3	2	2	3	10	3	6	2	12
Luminal B	3	2	0	0	0	1	1	2	0	3	3	3
Her-2	2	1	0	1	1	0	1	1	2	1	1	2
TN	3	1	0	1	1	2	0	2	2	5	2	4
Unknown	3	0	0	1	0	0	0	4	0	2	2	3
Clinical Stage												
0	3	0	0	1	0	0	0	1	0	1	0	3
I	5	2	0	2	0	2	2	7	0	3	1	7
II	8	2	0	1	2	2	2	7	6	9	6	12
III	1	0	0	2	2	1	1	3	1	4	3	2
IV	1	1	0	0	0	0	0	1	0	0	0	0
