# Supplementary figures and images for: Postpartum breast involution reveals regression of secretory lobules mediated by tissue-remodeling
Source: Breast Cancer Res. 2014 Mar 28;16(2):R31. doi: 10.1186/bcr3633 (PMC4053254; doi:10.1186/bcr3633)

# Supplementary Figure: 1S

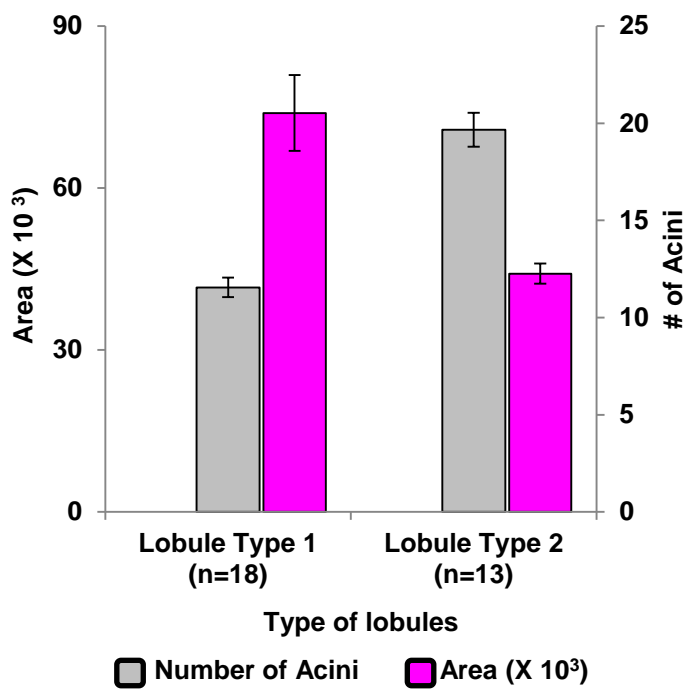

Supplement: Additional file 3: Figure S1 — Area-analysis cannot distinguish between lobular types 1 and 2. Some type 1 lobules can occupy larger areas when compared to smaller type 2 lobules due to variation in stromal to epithelial ratio. Eighteen type 1 lobules and 13 type 2 lobules were included in this analysis. All error bars represent standard error of the mean (SEM). [file bcr3633-S3.pdf]

# Supplementary Figure 2S

2S a

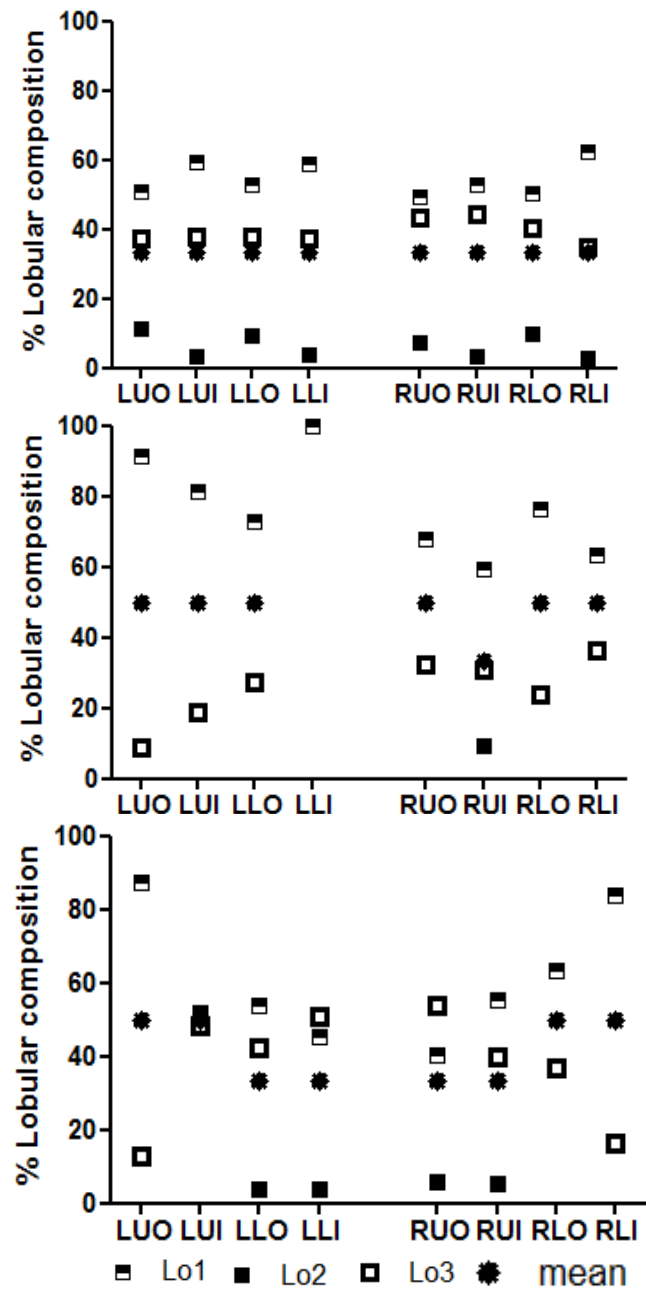

2S b

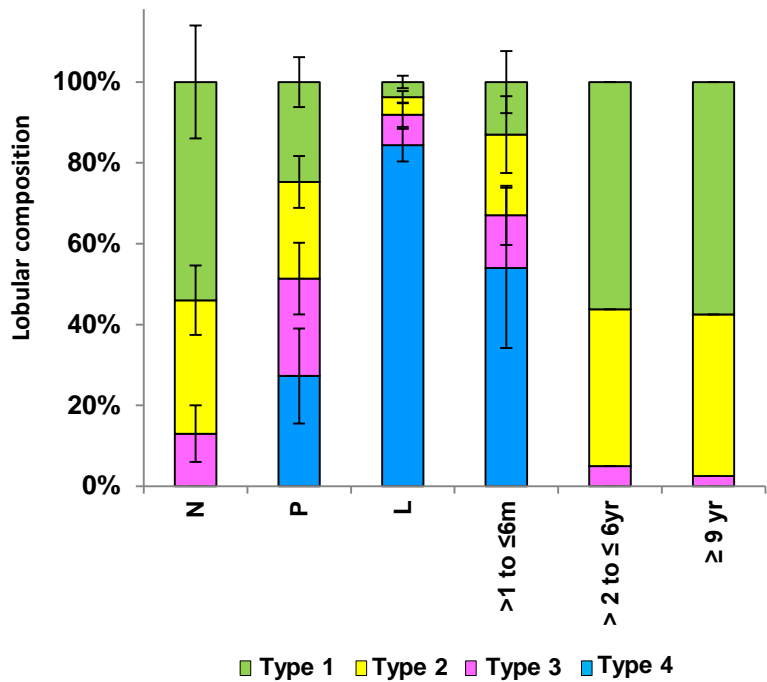

Supplement: Additional file 4: Figure S2 — Lobular composition by breast quadrant and cancer free cases. S2a: Lobular composition in individual breast quadrants obtained from bilateral mastectomy specimen of three women who were six to eight years postpartum. Each individual graph represents morphological variation in lobular composition for upper outer (UO), upper inner (UI), lower outer (LO) and lower inner (LI) quadrants of left (L) and right (R) breasts for each woman. S2b: Lobular composition across the reproductive cycle in women diagnosed with benign breast pathology. This cancer-free cohort of nulliparous (N) (n = 5), pregnant (P) (n = 11), lactation (L) (n = 8), >1 to ≤6 months (n = 5), >2 to ≤6 years (n = 1), and >9 years (n = 1) postpartum cases shows similar lobular composition as that of the total cohort (Figure 3B), suggesting that at the morphological level, postpartum involution is not significantly affected by the presence of adjacent tumor. All error bars represent standard error of the mean (SEM). [file bcr3633-S4.pdf]

Supplementary Figure 3S

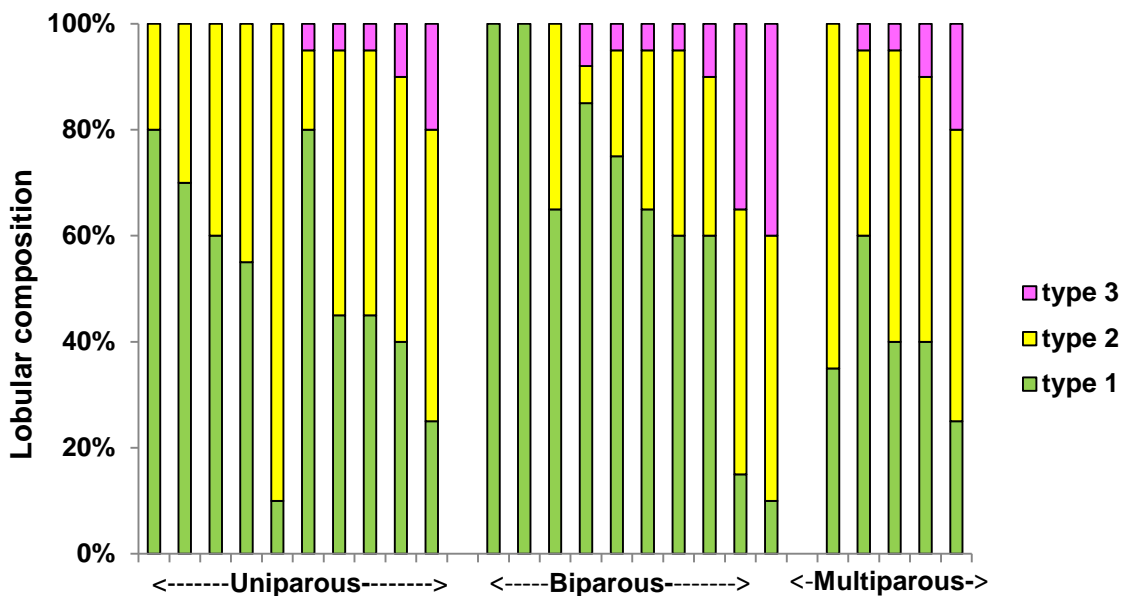

Supplement: Additional file 5: Figure S3 — Percent area composed of type 1, 2 and 3 lobules does not change with parity status. Percent of tissue composed of type 1, 2 and 3 lobules shows no correlation with the number of completed pregnancies, suggesting similar gland regression with each round of postpartum involution. [file bcr3633-S5.pdf]

# Supplementary Figure: 4S

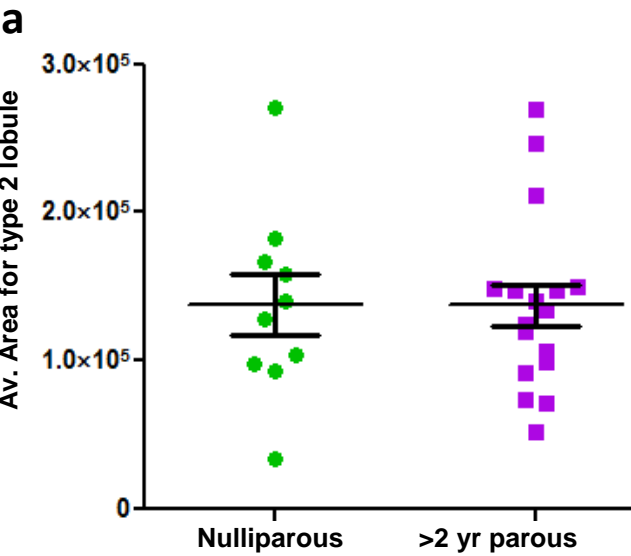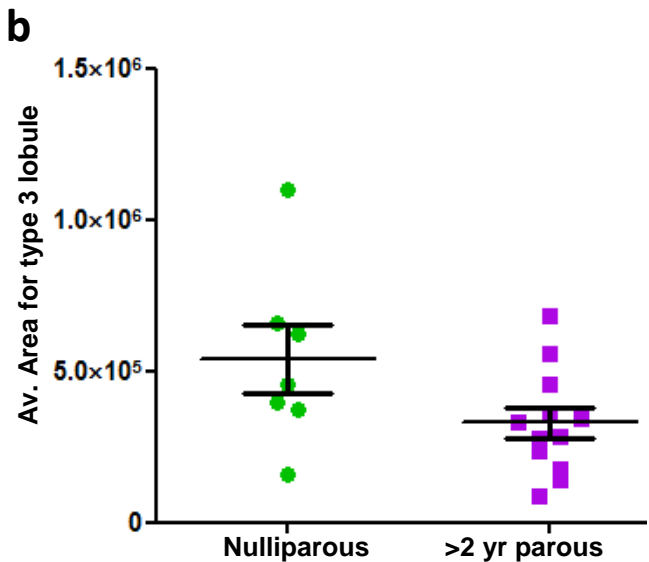

Supplement: Additional file 6: Figure S4 — Type 2 and 3 lobule size does not vary between nulliparous and parous cases. S4a. No differences are noted in average area of type 2 lobules between >2 years parous and nulliparous cases. S4b. The average area of type 3 lobules shows a trend towards the area being decreased in parous breast tissue compared to nulliparous breast issue (P = 0.06). All error bars represent standard error of the mean (SEM). [file bcr3633-S6.pdf]
